# Noninvasive pulmonary nodule characterization using transcutaneous bioconductance: Preliminary results of an observational study: Erratum

**DOI:** 10.1097/MD.0000000000017972

**Published:** 2019-11-11

In the article, “Noninvasive pulmonary nodule characterization using transcutaneous bioconductance: Preliminary results of an observational study”,^[1]^ which appeared in Volume 97, Issue 34 of *Medicine*, there is a correction.

The sentence “One of the challenges of this widespread screening are the number of false-positive lesions discovered, the NLST had a false-positive rate of 96.4% at baseline” should read “One of the challenges of this widespread screening is the high number of false-positive lesions discovered.”

## References

[1] Gariani J Martin SP Hachulla A Noninvasive pulmonary nodule characterization using transcutaneous bioconductance: Preliminary results of an observational study. *Medicine*. 2018 97: e11924. 30142805 10.1097/MD.0000000000011924 PMC6113006

